# The Georgia Medical Association

**Published:** 1900-05

**Authors:** 


### THE GEORGIA MEDICAL ASSOCIATION.

THE recent annual meeting of this Association, held in Atlanta on April 18th, 19th, and 20th, seemed to be one of eminent success. The program consisted of forty-nine papers, embracing nearly all the topics on medical subjects. Taking it on the whole, we do not think the papers showed that degree of merit which they should have possessed, a great many evidently having been written hurriedly without due study and preparation. Of course there were notable exceptions, and praise must be given to these. We do not think that the Georgia Medical Association has accomplished that amount of scientific work which should characterize its program at all the annual meetings, and we trust that greater efforts will be used in the future to accomplish such a result.

Some few new resolutions and by-laws were introduced at the meeting, which we think will prove altogether beneficial to the Association. Dr. Holt introduced an amendment to the by-laws by which the Association would revert to the old method of electing officers by nomination. His amendment had in view the appointment of a nominating committee, which should consist of one member from each county, and this committee should select the officers which were to be finally elected by the whole Association. By vote this measure was laid on the table until the next annual meeting. Another by-law introduced by the Board of Censors and ratified by the Association, was one compelling the Association to appoint one official stenographer from its own membership, provided such could be obtained. Dr. Claude Smith of Atlanta was elected to this position.

The Atlanta physicians endeavored to make the social side of the meeting one of pleasure to the visitors. On the first evening the president of the Association, Dr. F. W. McRae, gave a Bohemian Smoker at the Piedmont Driving Club. This was well attended, and proved a most enjoyable occasion. On the second evening a banquet was given at the Kimball House, in the large dining hall. Dr. Hunter Cooper, chairman of the Entertainment Committee, was master of cermonies. Toasts and speeches were delivered by Governor A. D. Candler, Hon. Clark Howell, Hon. F. H. Richardson, Dr. F. W. McRae.

Among the distinguished visitors who favored the Association with a paper was Dr. Wesley Boveé, of Washington, D. C.

The Association will meet next year in Augusta, Ga.

The following officers were elected:

President—Dr. S. C. Benedict, Athens, Ga.

Vice-President—Dr. R. M. Harbin, Rome, Ga.

Secretary—Dr. L. H. Jones, Atlanta, Ga.

Treasurer—Dr. E. C. Goodrich, Augusta, Ga.

---

IT is quite customary for the older surgeons to view with a manner not untinged with criticism the surgical aspirations of the younger members of the profession. They are quoted as saying that there is too much surgery being done by the inexperienced and inapt. When the citadels of life are invaded, it must be by a skilled craftsman, not a shrinking tyro ready to stampede at the first squirt of blood. With a self-consciousness little short of naive, they aver that no one is qualified to do serious and responsible surgery until he has gained that experience which gives confidence, dexterity, and technical *savior faire*. How inconsistent such expressions are and how unjust to the rising but obscure generation of medical workers is apparent on very slight reflection. How may surgical experience be gained? Is it intuition, or the result of work and observation? What was the evolutionary process which placed the critic upon his pedestal and gave him warrant to speak? Was it a gradation of slow ascents or a sudden development? Enough of these tedious and obvious platitudes. The truth is, the surgeon is born, not made. There are some who, by disposition and temperament, are especially adapted for taking on the heavy responsibility of surgical work. They are bold, self-reliant, cool, temperate and well-balanced men. Of all men in a community they are the most useful, the most indispensable. What a feeling of comfort and confidence is given to those who live remote from large cities by the knowledge that there is among them some one who is competent to meet the surgical emergencies which inevitably arise, and which without him would mean the premature termination of valuable lives.

Such men exist everywhere, but, contrasted with the bulk of commonplace humanity, they are not numerous. These are the men who do conscientious work. If they be not as expert as others who have served their novitiate in an ampler sphere, it is due to no fault of head or heart, but lack of opportunity. They approach the field of their battle with trepidation, but with a heart stiffened and a backbone braced by a sharp and near sense of the responsibility vested in them and of the necessity fcr action. They present quite a contrast to the nonchalance and easy indifference of the city hospital surgeon with his aseptic accoutrements and his horse, foot and dragoons of assistants. They suffer through external comparisons, but inwardly they are animated by the same spirit that led to the hewing of Agag.

The shaft of criticism glances off of these men. If there be too much surgery of a kind done, it is unjust to apply the assertion to these faithful, resourceful workers in the country and small towns. With them it is not a question of ambition, but of expediency, of grim, urgent, imperious necessity.

Further, if there is too much surgery, why is there such infinite subdivisions of the pedagogics of this subject? Every twig of the branches of surgery sags under the weight of some lecturer or instructor. If teaching is for the purpose of fitting the pupil for later practical application of theoretical knowledge, why should he be criticized if he carries out the legitimate conclusions in his practice? What is the purpose of the volumes of systematic works on surgery that cover the ground like autumn leaves? Are they written for those who already know, or to rid the author of his cerebral incrustations when they have become too bulky to remain in his skull, or are they intended as guides for those who do not know, but who are anxious to learn?

If there is too much surgery being done, it is done by those whose facility of opportunity and elevated position offer at once the occasion and the security from criticism that enables them to strain the quality of mercy out of essentially merciful work.

Spurred by ambition, forced by desire to retain an authoritative position to practice every new method, the temptation to perform unnecessary surgery becomes almost a coercion. To these the criticism applies with justice even though it be on the recoil.

---

THE *Brooklyn Medical Journal* calls attention to the report of the Pathological Society of that city on the common communion cup. It is no longer a theory but a well established fact that the use of the common communion cup is productive of many transmissible diseases, and is a menace to the health of the public. The above journal says there may be those who are skeptical . . . but "the actual finding of the bacilli of diphtheria in the cups used at a church service, after the use of which an epidemic of diphtheria broke out, would seem to be a demonstration which must satisfy every one. The possibilities, nay, the probabilities, are too horrible to contemplate." The journal further adds, "it is sincerely to be hoped that the influential body of scientific men who compose this Society (Pathological) will, by the broad dissemination of these valuable papers, so educate the churchgoers of this and other communities, that the adoption of the individual cup will be universal, and the further use of the common cup condemned as dangerous and disgusting."

---

"FOREST LODGE" SANATORIUM at Mt. Airy, Ga., is the first in this country to adopt the "Nordrach" or open-air treatment for the cure of consumption, and we commend the system as being scientific and according to authorities the best known.

---

A NEW medical organization consisting of genito-urinary specialists has been formed in New York, the office-bearers being—President, Dr. Ramon Guiteras, Professor of Genito-urinary Diseases at the New York Post-Graduate School and Hospital; first vice-president, Dr. Winfield Ayers, Bellevue Hospital; second vice-president, Dr. Otis K. Newell, formerly of the Harvard Medical School, now of New York; treasurer, Dr. George W. Blanchard; secretary, Dr. A. D. Mabie; corresponding secretary and stenographer, Mr. Samuel Bennett, 161 Garfield Place, Brooklyn, from whom information as to terms of membership, etc., may be obtained. Correspondence is invited with specialists in other parts of the country and abroad.

